# The association between, depression, anxiety, and mortality in older people across eight low‐ and middle‐income countries: Results from the 10/66 cohort study

**DOI:** 10.1002/gps.5211

**Published:** 2019-10-24

**Authors:** Yu‐Tzu Wu, Carolina Kralj, Daisy Acosta, Mariella Guerra, Yueqin Huang, Amuthavalli T. Jotheeswaran, Ivonne Z. Jimenez‐Velazquez, Zhaorui Liu, Juan J. Llibre Rodriguez, Aquiles Salas, Ana Luisa Sosa, Rasha Alkholy, Martin Prince, A. Matthew Prina

**Affiliations:** ^1^ King's College London, Social Epidemiology Research Group, Health Service and Population Research Institute of Psychiatry, Psychology & Neuroscience London UK; ^2^ South London and Maudsley NHS Foundation Trust London UK; ^3^ Internal Medicine Department, Geriatric Section Universidad Nacional Pedro Henriquez Ureña (UNPHU) Santo Domingo Dominican Republic; ^4^ Psychogeriatric Unit National Institute of Mental Health Honorio Delgado Hideyo Noguchi, Lima, Peru and Centro de la Memoria y Desordenes Relacionados Lima Perú; ^5^ Peking University, Institute of Mental Health Beijing China; ^6^ Department of Ageing and Life Course World Health Organization Geneva Switzerland; ^7^ Internal Medicine Department, Geriatrics Program, School of Medicine, Medical Sciences Campus University of Puerto Rico San Juan Puerto Rico; ^8^ Facultad de Medicina Finlay‐Albarran Medical University of Havana Havana Cuba; ^9^ Medicine Department, Caracas University Hospital, Faculty of Medicine Universidad Central de Venezuela Caracas Venezuela; ^10^ National Institute of Neurology and Neurosurgery of Mexico National Autonomous University of Mexico Mexico City Mexico; ^11^ King's College London Global Health Institute London UK

**Keywords:** anxiety, common mental disorders, depression, epidemiology, mortality

## Abstract

**Objectives:**

Depression and anxiety are common mental disorders in later life. Few population‐based studies have investigated their potential impacts on mortality in low‐ and middle‐income countries (LMICs). The aim of this study is to examine the associations between depression, anxiety, their comorbidity, and mortality in later life using a population‐based cohort study across eight LMICs.

**Methods:**

This analysis was based on the 10/66 cohort study including 15 991 people aged 65 years or above in Cuba, Dominican Republic, Venezuela, Mexico, Peru, Puerto Rico, China, and India, with an average follow‐up time of 3.9 years. Subthreshold and clinical levels of depression were determined using EURO‐D and ICD‐10 criteria, and anxiety was based on Geriatric Mental State (GMS)–Automated Geriatric Examination for Computer Assisted Taxonomy (AGECAT). Cox proportional hazard modelling was used to estimate how having depression, anxiety, or both was associated with mortality adjusting for sociodemographic and health factors.

**Results:**

Participants with clinical depression (hazard ratio [HR]: 1.45; 95% CI, 1.24‐1.70) and subthreshold anxiety (HR: 1.26; 95% CI, 1.15‐1.38) had higher risk of mortality than those without the conditions after adjusting for sociodemographic factors and health conditions. Comorbidity of depression and anxiety was associated with a 30% increased risk of mortality but the effect sizes varied across countries (Higgins I^2^ = 58.8%), with the strongest association in India (HR: 1.99; 95% CI, 1.21‐3.27).

**Conclusions:**

Depression and anxiety appear to be associated with mortality in older people living in LMICs. Variation in effect sizes may indicate different barriers to health service access across countries. Future studies may investigate underlying mechanisms and identify potential interventions to reduce the impact of common mental disorders.

**Key points**

- A small number of population‐based studies have investigated the association between depression and mortality in low‐ and middle‐income countries but few have examined the potential impacts of depression, anxiety, and their comorbidity on mortality in later life.
- Using a population‐based cohort study of 15 991 older people from eight low‐ and middle‐income countries, this study reports that depression and anxiety were associated with increased mortality risk in later life and their comorbidity was related to a 30% increased mortality risk.
- The effect sizes were found to vary across countries. This may indicate different barriers to health service access in low‐ and middle‐income countries.

## 1 INTRODUCTION

Depression and anxiety are common mental disorders in later life. It is estimated that 10% to 20% of older people are affected by depression, which is frequently comorbid with anxiety disorders.^1^, ^2^, ^3^, ^4^ These two conditions have been strongly associated with increased risk of disability and mortality in older age.^5^, ^6^, ^7^ According to the World Health Organisation, depression accounts 5.7% years lived with disability (YLDs) among people aged 60 years or above^8^ and these two conditions together contributed to 55% of disability‐adjusted life years attributable to mental and substance use disorders.^9^ In addition, subthreshold conditions, which are found to be two to three times more prevalent than clinical depression and anxiety,^10^, ^11^ have been suggested to have a comparable impact on increased risk of mortality.^12^

The associations between depression, anxiety, and mortality are well‐established in the literature,^13^ and some possible mechanisms have been linked to changes in health behaviours, neuroendocrine and immune systems, and increased risk of cardiovascular diseases and suicide.^14^, ^15^, ^16^ However, most existing studies are based on study populations from high‐income countries.^7^, ^13^ Although biological mechanisms could be similar across countries, the assessment and diagnosis of mental disorders can be more complicated in low‐ and middle‐income countries (LMICs) due to cultural variation.^17^ Stigma of mental illness and limited access to health care in LMICs might affect help‐seeking behaviour and lead to poor prognosis of depression and anxiety disorders.^18^, ^19^, ^20^ Thus, common mental disorders may have different associations with mortality among older people who do not live in high‐income settings.

Several studies from LMICs have investigated the association between depression and mortality using patient records or hospital data.^7^ Given cultural variation and the complexity of assessment and diagnosis, these results based on highly selected populations might be biased and there is limited evidence from population‐based studies. A recent systematic review which focused on depression and mortality in older people identified 10 population‐based studies from LMICs.^21^ Although the pooled effect size was similar to high‐income countries, the high heterogeneity reported in the review might be attributed to variation in diagnostic methods and study design.^21^ Furthermore, to our knowledge, no population‐based study from LMICs has assessed the association between anxiety disorder, comorbid anxiety and depression, and mortality.

The aim of this study is to investigate the potential impact of depression, anxiety, and mortality using a population‐based cohort study of older people across eight LMICs in Latin America and Asia. The analysis examined individual associations for clinical and subthreshold levels of depression and anxiety as well as comorbidity of these two conditions.

## 2 MATERIALS AND METHODS

### 2.1 Study design

The 10/66 Dementia Research Group carried out surveys of older people aged 65 years and over living in 11 catchment areas across eight LMICs including China, Cuba, Dominican Republic, India, Mexico, Peru, Puerto Rico, and Venezuela. China, Mexico, and Peru had one urban and one rural site, whereas the other countries only included an urban site. Each site covered a catchment area which had well‐defined boundaries and contributed between 1000 and 3000 participants to the study. The selection of catchment areas was based on accessibility, and the research networks and areas with high‐income residents were avoided. All households in the catchment areas were visited by interviewers to identify eligible participants aged 65 years or above. The baseline surveys were conducted between 2003 and 2005 for all sites apart from Puerto Rico, where data were collected from 2007. Response rates were excellent across all sites (72%‐98%). A full follow‐up, which also included a mortality sweep to determine vital status, was carried out 3 to 5 years after the baseline and date of death of those deceased were recorded. Informed consent was obtained from all participants. The 10/66 study was approved by local ethical committees and by the King's College London research ethics committee. Full details of the protocol and the cohort are available elsewhere.^22^, ^23^, ^24^

This study excluded participants from the rural site of India (N = 999), as the follow up was not conducted in this area. This left 15 991 participants from all eight countries. Ethical approval was not required, as this secondary data analysis did not include data on individual patients.

### 2.2 Measurements

A full assessment of each participant took place within the household and lasted between 2 and 3 hours. Each standardised assessment was translated, back‐translated, and adapted into the different languages of each site. The assessment included a structured interview, an informant interview, and a comprehensive physical examination.

The Geriatric Mental State (GMS) examination and its computerised algorithm Automated Geriatric Examination for Computer Assisted Taxonomy (AGECAT) were administered to participants to assess their mental health states. Anxiety was defined as reaching level 3 in the GMS‐AGECAT stage 1 anxiety axis, as this threshold usually reflects a state which would necessitate professional intervention.^4^ Levels 1 and 2 are considered as subthreshold cases of anxiety. Depression was determined using EURO‐D^25^, ^26^ and ICD‐10 criteria generated using specific GMS algorithms, as used in previous research in those settings.^4^, ^27^ Cases of depression were defined as those having ICD‐10 depression, whereas subthreshold cases were those having scored 4 or 5 on the EURO‐D scale, but not meeting ICD‐10 depression criteria. Procedures to select the optimal cutpoint of 4 or 5 on the EURO‐D scale have been reported in the EURO‐D validation paper.^25^ To increase statistical power, cases and subthreshold cases of anxiety and depression were combined to identify participants with comorbidity of any levels of symptoms and those with depression or anxiety alone.

Other measurements relevant to this study included age, gender, education (none/did not complete primary, completed primary, secondary, tertiary), number of limiting physical impairments (no illnesses, one or two illnesses, three or more), number of assets within the household, food insecurity, and dementia. Dementia was defined as 10/66 dementia diagnosis adjusted for education, and further information on this measure is available elsewhere.^28^

### 2.3 Statistical analysis

Kaplan‐Meier failure curves were conducted to estimate cumulative incidence of mortality by depression, anxiety, and comorbidity of the two conditions, and log‐rank test was used to examine differences across these groups. Schoenfeld Residual tests were used to test the proportional hazard assumption. Cox proportional hazard modelling was used to examine the associations between mortality, depression, and anxiety levels and their comorbid conditions. Three types of models were conducted including an unadjusted model (model 1), a model adjusted for age and gender (model 2), and a fully adjusted model for all covariates (model 3). All models were estimated in each country and pooled estimates for the overall 10/66 cohort were calculated using fixed‐effect meta‐analysis, and reported with a measure of their heterogeneity (Higgins I^2^).^29^ A fixed‐effect meta‐analysis was considered to be more appropriate than a random‐effect meta‐analysis, as the aim here was to summarise estimates from all 10/66 sites rather than make inferences to other hypothetical populations. This analytical approach has been widely applied to most studies using the 10/66 cohort.^4^, ^24^

The method of inverse probability weighting was used to account for the 13% lost to follow up (N = 2072).^30^ The weights were generated based on all variables in the fully adjusted model (age, gender, education, depressive level, anxiety level, dementia, physical impairment, assets, and food insecurity) and country, which is associated with the probability of lost to follow up and prevalence of depression and anxiety status. These weights were applied to all cox regression models. The total number of missing covariates was small (N = 219) and considered to have a limited impact on estimates. An additional group was generated to include those with missing covariates in the analysis. All analyses were conducted using Stata 15.0. A STROBE checklist is provided in Data S1.

## 3 RESULTS

Table 1 reports descriptive information on the study population. The mean age was 74.3 years (SD = 7.1) and 63% were women. Over 40% did not have any education or complete primary school. Among 15 991 participants, 16% (N = 2591) were dead at the follow up wave and 13% were lost to follow up. The prevalence of depression was 11.6% (N = 1858) for subthreshold and 5.1% (N = 817) for clinical levels. For anxiety, the prevalence was 38.1% (N = 6084) for subthreshold and 5.1% (N = 812) for clinical levels. The comorbidity of any depression and anxiety levels was 14.8% (N = 2366). Only 309 (1.9%) had depression alone, and 4530 (28.3%) had anxiety alone.

**Table 1:** Sociodemographic and health factors of the study population (N = 15 991)

	Cuba	Dominican Republic	Mexico	Peru	Puerto Rico	Venezuela	China	India	Total
N	2937	2009	2002	1932	2002	1961	2162	986	15 991
Age: mean (SD)	75.1 (7.0)	75.3 (7.5)	74.3 (6.7)	74.8 (7.4)	76.4 (7.4)	72.5 (6.9)	73.2 (6.1)	71.3 (6.1)	74.3 (7.1)
Women: N (%)	1909 (65.0)	1325 (66.0)	1267 (63.3)	1182 (61.2)	1347 (67.3)	1249 (63.7)	1217 (56.3)	568 (57.6)	10 064 (62.9)
Low education: N (%) (missing = 87)	726 (24.8)	1413 (71.0)	1417 (70.9)	351 (18.3)	461 (23.1)	599 (31.2)	1078 (49.9)	653 (66.3)	6698 (42.1)
3+ physical impairments: N (%) (missing = 41)	292 (10.0)	464 (23.1)	343 (17.1)	264 (13.7)	429 (21.4)	488 (25.3)	247 (11.4)	40 (4.1)	2567 (16.1)
Dementia: N (%) (missing = 25)	315 (10.8)	235 (11.7)	170 (8.5)	164 (8.5.)	233 (11.7)	137 (7.0)	137 (6.3)	75 (7.6)	1466 (9.2)
Food insecurity: N (%) (missing = 179)	140 (4.8)	240 (12.1)	124 (6.2)	137 (7.2)	32 (1.6)	111 (6.0)	12 (0.6)	205 (20.9)	1001 (6.3)
Number of assets: median (IQR) (missing = 10)	6.0 (1.0)	5.0 (2.0)	6.0 (2.0)	6.0 (0.0)	7.0 (1.0)	6.0 (1.0)	6.0 (1.0)	4.0 (2.0)	6.0 (1.0)
Lost to follow up: N (%)	309 (10.5)	305 (15.2)	158 (7.9)	181 (9.4)	431 (21.5)	266 (13.6)	171 (7.9)	251 (25.5)	2072 (13.0)
Dead: N (%)	607 (23.1)	467 (27.4)	209 (11.3)	152 (8.7)	297 (18.9)	198 (11.7)	515 (25.9)	146 (14.8)	2591 (16.3)
Years of follow up: median (IQR)	4.2 (1.5)	5.0 (1.5)	3.0 (1.2)	3.0 (1.2)	4.3 (1.0)	4.2 (0.8)	4.9 (0.8)	2.9 (1.1)	4.0 (1.8)

*Note*. Low education: None or did not complete primary education.

Abbreviation: IQR, interquartile range.

Table 2 shows the associations between depression, anxiety, and mortality. Compared with those without depression, people with subthreshold (hazard ratio [HR]: 1.21; 95% CI, 1.07‐1.37) and clinical depression (HR: 1.82; 95% CI, 1.57‐2.11) had higher risk of mortality in the pooled estimates. After adjusting for sociodemographic factors and health conditions, the effect size of subthreshold depression largely attenuated (HR: 1.06; 95% CI, 0.93‐1.22) but clinical depression remained to be associated with mortality (HR: 1.45; 95% CI, 1.24‐1.70). For anxiety, both subthreshold (HR: 1.34; 95% CI, 1.23‐1.46) and clinical levels (HR: 1.35; 95% CI, 1.12‐1.63) were associated with 30% higher risk of mortality and the effect sizes slightly reduced after adjustment. Apart from clinical depression, the heterogeneity across countries was high with a range of Higgins I^2^ between 53% and 56.8%.

**Table 2:** Hazard ratios for mortality by depressive and anxiety levels (subthreshold or case vs free)

		Depression			Anxiety		
		Model 1	Model 2	Model 3	Model 1	Model 2	Model 3
Cuba	Subthreshold	1.38 (1.09‐1.73)	1.40 (1.10‐1.76)	1.24 (0.97‐1.58)	1.30 (1.10‐1.53)	1.36 (1.15‐1.62)	1.10 (0.92‐1.31)
	Case	1.71 (1.27‐2.31)	1.98 (1.47‐2.66)	1.59 (1.17‐2.17)	1.02 (0.68‐1.52)	1.43 (0.97‐2.11)	1.05 (0.70‐1.58)
Dominican Republic	Subthreshold	1.37 (1.06‐1.77)	1.25 (0.96‐1.64)	1.18 (0.90‐1.55)	1.49 (1.22‐1.82)	1.37 (1.12‐1.68)	1.25 (1.01‐1.55)
	Case	1.68 (1.32‐2.13)	1.61 (1.27‐2.03)	1.37 (1.06‐1.78)	1.70 (1.23‐2.35)	1.74 (1.24‐2.42)	1.31 (0.92‐1.88)
Peru	Subthreshold	0.61 (0.34‐1.09)	0.64 (0.36‐1.13)	0.59 (0.33‐1.05)	0.86 (0.62‐1.19)	0.95 (0.69‐1.32)	0.96 (0.68‐1.35)
	Case	1.92 (1.12‐3.30)	1.92 (1.12‐3.30)	1.40 (0.76‐2.58)	0.56 (0.26‐1.23)	0.64 (0.29‐1.41)	0.47 (0.21‐1.06)
Venezuela	Subthreshold	1.16 (0.77‐1.73)	1.27 (0.84‐1.92)	1.06 (0.67‐1.66)	1.48 (1.09‐2.02)	1.51 (1.10‐2.09)	1.38 (0.98‐1.92)
	Case	2.71 (1.75‐4.21)	2.77 (1.81‐4.24)	1.94 (1.17‐3.22)	2.55 (1.64‐3.95)	2.66 (1.70‐4.16)	1.91 (1.11‐3.27)
Mexico	Subthreshold	0.91 (0.60‐1.37)	0.92 (0.61‐1.39)	0.78 (0.50‐1.22)	1.56 (1.18‐2.05)	1.58 (1.20‐2.08)	1.47 (1.10‐1.97)
	Case	1.42 (0.81‐2.48)	1.38 (0.78‐2.45)	1.10 (0.62‐1.97)	0.89 (0.45‐1.76)	0.99 (0.49‐2.00)	0.81 (0.39‐1.68)
Puerto Rico	Subthreshold	0.61 (0.36‐1.02)	0.67 (0.39‐1.15)	0.52 (0.30‐0.91)	0.94 (0.74‐1.20)	1.14 (0.89‐1.45)	1.06 (0.83‐1.37)
	Case	1.23 (0.57‐2.66)	1.40 (0.66‐2.98)	1.01 (0.45‐2.26)	0.72 (0.37‐1.40)	1.16 (0.60‐2.27)	1.03 (0.49‐2.16)
China	Subthreshold	1.41 (0.68‐2.94)	1.21 (0.59‐2.46)	1.06 (0.51‐2.19)	1.70 (1.30‐2.24)	1.74 (1.35‐2.24)	1.68 (1.29‐2.18)
	Case	3.08 (1.43‐6.60)	2.88 (1.20‐6.90)	1.49 (0.74‐3.01)	2.07 (0.40‐10.64)	4.16 (0.88‐19.66)	4.07 (1.39‐11.89)
India	Subthreshold	1.55 (1.07‐2.22)	1.66 (1.14‐2.40)	1.41 (0.93‐2.14)	1.81 (1.31‐2.52)	1.88 (1.35‐2.62)	1.77 (1.25‐2.50)
	Case	2.11 (1.04‐4.25)	2.10 (1.00‐4.43)	1.54 (0.64‐3.70)	1.13 (0.33‐3.80)	1.24 (0.35‐4.33)	1.12 (0.30‐4.14)
**Pooled estimate**	**Subthreshold**	**1.21 (1.07‐1.37)**	**1.21 (1.06‐1.38)**	**1.06 (0.93‐1.22)**	**1.34 (1.23‐1.46)**	**1.40 (1.28‐1.52)**	**1.26 (1.15‐1.38)**
	**I** ^**2**^	60.9	54.6	56.4	71.2	53.9	56.8
	**Case**	**1.82 (1.57‐2.11)**	**1.85 (1.60‐2.14)**	**1.45 (1.24‐1.70)**	**1.35 (1.12‐1.63)**	**1.58 (1.30‐1.91)**	**1.20 (0.98‐1.48)**
	**I** ^**2**^	0.0	8.5	0.0	68.2	53.2	53.0

*Note*. Model 1: unadjusted. Model 2: adjusted for age and gender. Model 3: adjusted for age, gender, education, assets, food insecurity, limiting physical impairment, and dementia. Bold: Pooled estimates across the eight countries

**Figure 1:**
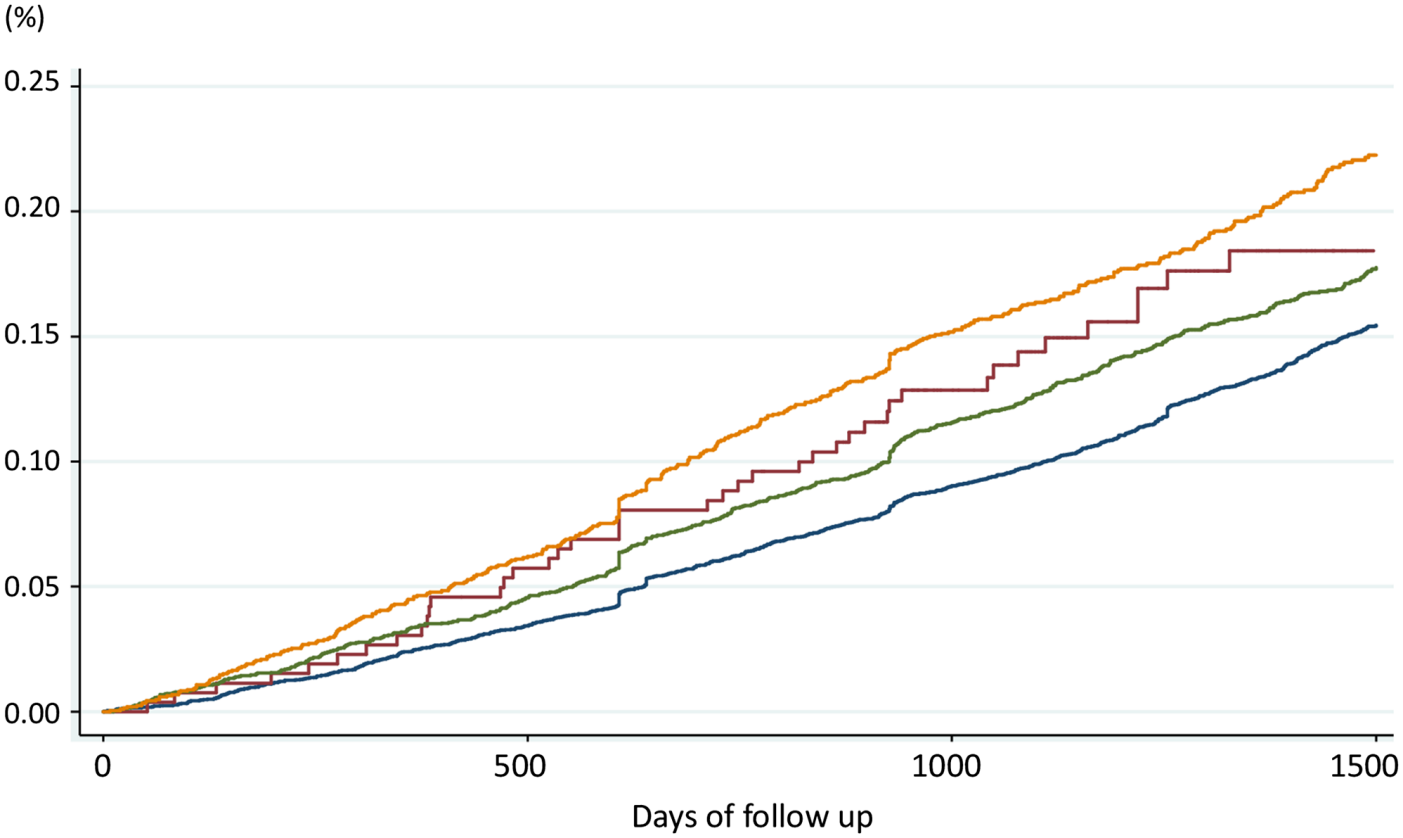
Kaplan‐Meier failure curves of mortality by comorbid conditions of depression and anxiety (blue, none; green, anxiety alone; red, depression alone; yellow, comorbidity of depression and anxiety) [Colour figure can be viewed at http://wileyonlinelibrary.com]

Figure 1 shows Kaplan‐Meier failure curves by those with no conditions, depression alone, anxiety alone, and comorbidity of the two conditions. Among the four groups, participants with comorbidity of depression and anxiety had the highest incidence of mortality. The results of Cox regression modelling are reported in Table 3. Anxiety alone was associated with a 24% higher risk of mortality (HR: 1.24; 95% CI, 1.13‐1.37) after adjusting for all covariates. Participants with comorbid depression and anxiety had a 30% higher risk of mortality (HR: 1.29; 95% CI, 1.14‐1.47) than those without any depression and anxiety, and the effect sizes varied across countries (Higgns I^2^ = 58.8%). More detailed results of fully adjusted models are reported in Tables S1 to S3.

**Table 3:** Hazard ratios for mortality by comorbidity of depression and anxiety (depression alone or anxiety alone vs free)

		Model 1	Model 2	Model 3
Cuba	Dep alone	1.53 (0.89‐2.65)	1.39 (0.83‐2.33)	1.34 (0.80‐2.24)
	Anx alone	1.16 (0.96‐1.39)	1.23 (1.02‐1.49)	1.01 (0.83‐1.23)
	Comorbidity	1.56 (1.26‐1.93)	1.73 (1.38‐2.16)	1.35 (1.07‐1.71)
Dominican Republic	Dep alone	0.66 (0.28‐1.58)	0.62 (0.25‐1.55)	0.65 (0.26‐1.63)
	Anx alone	1.28 (1.02‐1.60)	1.21 (0.97‐1.51)	1.12 (0.89‐1.42)
	Comorbidity	1.82 (1.45‐2.28)	1.66 (1.31‐2.10)	1.42 (1.10‐1.84)
Peru	Dep alone	1.11 (0.34‐3.65)	1.37 (0.40‐4.64)	1.45 (0.42‐5.00)
	Anx alone	0.80 (0.56‐1.15)	0.92 (0.64‐1.31)	0.96 (0.65‐1.40)
	Comorbidity	0.86 (0.55‐1.36)	0.93 (0.58‐1.47)	0.79 (0.49‐1.26)
Venezuela	Dep alone	0.95 (0.22‐4.03)	0.95 (0.22‐4.09)	0.87 (0.22‐3.38)
	Anx alone	1.47 (1.07‐2.04)	1.46 (1.04‐2.05)	1.36 (0.96‐1.92)
	Comorbidity	1.95 (1.35‐2.81)	2.13 (1.45‐3.12)	1.61 (1.02‐2.54)
Mexico	Dep alone	1.59 (0.68‐3.75)	1.67 (0.71‐3.93)	1.48 (0.61‐3.57)
	Anx alone	1.65 (1.22‐2.22)	1.71 (1.27‐2.30)	1.63 (1.20‐2.21)
	Comorbidity	1.24 (0.84‐1.85)	1.26 (0.85‐1.88)	1.06 (0.68‐1.63)
Puerto Rico	Dep alone	0.58 (0.07‐4.56)	0.44 (0.05‐4.09)	0.38 (0.04‐3.75)
	Anx alone	0.97 (0.75‐1.25)	1.20 (0.93‐1.55)	1.20 (0.92‐1.56)
	Comorbidity	0.73 (0.46‐1.14)	0.90 (0.57‐1.43)	0.68 (0.42‐1.11)
China	Dep alone	1.36 (0.42‐4.44)	0.91 (0.28‐2.97)	0.75 (0.23‐2.49)
	Anx alone	1.64 (1.22‐2.21)	1.70 (1.30‐2.22)	1.70 (1.29‐2.25)
	Comorbidity	2.17 (1.18‐3.99)	2.19 (1.15‐4.16)	1.67 (0.98‐2.84)
India	Dep alone	1.21 (0.61‐2.38)	1.38 (0.70‐2.70)	1.24 (0.61‐2.53)
	Anx alone	1.55 (1.03‐2.33)	1.64 (1.09‐2.47)	1.68 (1.11‐2.52)
	Comorbidity	2.21 (1.47‐3.31)	2.35 (1.55‐3.58)	1.99 (1.21‐3.27)
**Pooled Estimate (fixed)**	**Dep alone**	**1.22 (0.90‐1.67)** **I** ^**2**^ **= 0.0%**	**1.21 (0.89, 1.65)** **I** ^**2**^ **= 0.0%**	**1.13 (0.83‐1.55)** **I** ^**2**^ **= 0.0%**
	**Anx alone**	**1.25 (1.14‐1.38)** **I** ^**2**^ **= 63.0%**	**1.33 (1.21, 1.46)** **I** ^**2**^ **= 47.5%**	**1.24 (1.13‐1.37)** **I** ^**2**^ **= 59.0%**
	**Comorbidity**	**1.55 (1.38‐1.74)** **I** ^**2**^ **= 72.9%**	**1.61 (1.43, 1.82)** **I** ^**2**^ **= 64.1%**	**1.29 (1.14‐1.47)** **I** ^**2**^ **= 58.8%**

*Note*. Model 1: unadjusted. Model 2: adjusted for age and gender. Model 3: adjusted for age, gender, education, assets, food insecurity, limiting physical impairment and dementia.

Abbreviations: Anx, anxiety; Dep, depression. Bold: Pooled estimates across the eight countries

## 4 DISCUSSION

### 4.1 Main findings

This study investigated the impact of depression and anxiety disorders on mortality risk in older people from eight LMICs. The results suggest that these two common mental disorders, in particular clinical depression and any levels of anxiety disorders, were associated with increased risk of mortality in later life. Comorbidity of depression and anxiety was associated with a 30% higher risk of mortality after adjusting for sociodemographic factors and health conditions. High variation in effect sizes were found across countries.

### 4.2 Strengths and limitations

This study was based on a population‐based cohort recruiting older people from various settings across several LMICs. Structured interviews were conducted to assess depression and anxiety levels in different contexts and this provided comparable information on common mental disorders. The analysis identified those with subthreshold and clinical levels of depression and anxiety and considered severity levels of these common mental disorders. However, there are some limitations. Although the 10/66 study population was selected to be as representative as possible of the general population in each country, it is unlikely to be a nationally representative sample and this might affect generalisability of the results. The study included nearly 16 000 participants, but it was difficult to formally test variations across countries due to limited statistical power. Since most participants with depression (88%) also had anxiety disorders, there was limited statistical power to examine the effect sizes of depression alone. The attrition rate was considered to be low (13%) in general but varied across sites. In particular, the rural site of India was not included in the follow up surveys. Although inverse probability weighting was used to account for lost to follow up, the results could be underestimated if the associations between depression, anxiety, and mortality were stronger in those who did not attend the follow up waves. Some factors related to physical health and mortality, such as smoking, alcohol consumption, and obesity, were not adjusted in the models and might attenuate the strength of associations between common mental disorders and mortality. However, the measure for limiting physical impairments included a wide range of common health problems, such as arthritis/rheumatism, high blood pressure, and heart trouble/angina, which can be consequences of unhealthy lifestyle.

### 4.3 Interpretation of results

The results of this study suggest a negative impact of depression and anxiety on mortality in older people living in LMICs. This corresponds to the majority of existing studies in high‐income countries and a recent review on LMICs.^21^ A meta‐analysis on mortality rates in clinical and subthreshold depression has summarised 22 studies and suggested a 60% increased mortality risk for clinical cases and 30% for subthreshold levels.^12^ The review focusing on 10 studies in LMICs also suggested a 60% increased risk of mortality for depression.^21^ These effect sizes were slightly stronger than the estimates reported in this study, and the difference could be related to variation in covariate adjustment. The associations between anxiety disorders and mortality seem to be inconclusive and the estimates vary across different systematic reviews.^13^, ^31^ This study found an approximate 25% increased risk of mortality in those with anxiety disorders. Although variations in research methods and measurements for anxiety disorders can contribute to different findings, the impact of anxiety on mortality could be particularly important in older people living in some LMICs, where recognition and treatment of mental conditions can still be limited.^18^

This study suggested that comorbidity of depression and anxiety was associated with even higher risk of mortality than depression or anxiety alone. However, this addictive effect was not found in the Health Study of Nord‐Trondelag County (HUNT), a population‐based study including over 60 000 adults in Norway.^32^ The findings suggest that case‐level depression was associated with a 30% increased risk of mortality after adjusting for sociodemographic factors, physical health status, and lifestyle factors. However, the effect size of comorbid case‐level anxiety and depression was smaller than depression alone. A possible explanation proposed in the publication was that people with moderate levels of anxiety might have better help‐seeking behaviour and adherence to treatment than those with low trait anxiety. This may not be applicable to the settings of our research, where access to services is still limited, and which could consequently explain why comorbidity of depression and anxiety had a stronger impact than single conditions. It also reflects previous research from our group, showing that comorbid anxiety and depression was associated with higher levels of disability than having one condition alone.^4^

Mechanisms between common mental disorders and mortality in later life have been largely attributed to cause‐specific mortality of cardiovascular diseases, suicides, and death related to alcohol and substance use.^14^, ^15^ Depression and anxiety can also act as risk factors for noncompliance with medical treatments, and this might increase risk of mortality.^33^ These mechanisms may also play an important role in LMICs, where the burden of noncommunicable disease and mental disorders is predicted to increase due to population ageing, urbanisation, and changes in lifestyle.^34^ In addition, stigma and misunderstanding of mental disorders have been recognised to be an important issue in LMICs and may affect help‐seeking behaviour and lead to under‐diagnosis of common mental disorders in later life.^19^, ^20^ These societal factors might be barriers to health services and contribute to the heterogeneity of effect sizes found in different countries.

### 4.4 Clinical implications and future research directions

Since depression and anxiety are treatable conditions, access to mental health services and provision of medical treatments may be effective to reduce the impact of common mental disorders in later life. Although recognition of common mental disorders in later life might be improved through trainings of clinicians and health professionals, additional resources are needed to improve policy and infrastructure for mental health services in LMICs and address stigma and misunderstanding of mental disorders.^18^

In addition to the link between depression and cardiovascular diseases, future research may investigate cause of mortality and identify specific pathways in the context of LMICs. Some contextual factors in different countries, such as diagnosis, accessibility to mental health services, and treatment availability, may play an important role in modifying the impact of common mental disorders in later life and needs to be incorporated in future research. Cross‐country studies using consistent research methods and measurements for mental disorders are needed to provide comparable estimates across different studies populations. The development of possible interventions may focus on improving access to medical services and treatments of depression and anxiety and addressing determinants and risk factors for mental disorders.

In conclusion, this study provides empirical evidence on the substantial impact of depression, anxiety, and their comorbidity on mortality in older people living in LMICs. Different effect sizes across countries may indicate potential barriers to access to health care. Future research may investigate underlying mechanisms and develop possible interventions to improve access to mental health services and treatments of mental disorders in LMICs.

## CONFLICT OF INTERESTS

All authors declared no conflicts of interest.

## Supporting information

Data S1. Data supplementary

**Table S1:** Fully adjusted model of the association between depression and mortality
**Table S2:** Fully adjusted model of the association between anxiety and mortality
**Table S3:** Fully adjusted model of the association between depression, anxiety and mortality

## Data Availability

Data from the 10/66 study are available on request to the research team and details for potential applicants are contained in the project website (https://www.alz.co.uk/1066/).
