# Supplementary material for: The association between, depression, anxiety, and mortality in older people across eight low‐ and middle‐income countries: Results from the 10/66 cohort study
Source: Int J Geriatr Psychiatry. 2019 Oct 24;35(1):29–36. doi: 10.1002/gps.5211 (PMC6916169; doi:10.1002/gps.5211)
Supplement: Supplementary file 2 — Table S1: Fully adjusted model of the association between depression and mortality Table S2: Fully adjusted model of the association between anxiety and mortality Table S3: Fully adjusted model of the association between depression, anxiety and mortality [file GPS-35-29-s002.docx]

**Supporting Information**

**Table S1:** Fully adjusted model of the association between depression and mortality

|  | Cuba | Dominican Republic | Peru | Venezuela | Mexico | Puerto Rico | China | India |
| --- | --- | --- | --- | --- | --- | --- | --- | --- |
| Depression (ref: none) |  |  |  |  |  |  |  |  |
| Sub-threshold | 1.24 (0.97, 1.58) | 1.18 (0.90, 1.55) | 0.59 (0.33, 1.05) | 1.06 (0.67, 1.66) | 0.78 (0.50, 1.22) | 0.52 (0.30, 0.91) | 1.06 (0.51, 2.19) | 1.41 (0.93, 2.14) |
| Case | 1.59 (1.17, 2.17) | 1.37 (1.06, 1.78) | 1.40 (0.76, 2.58) | 1.94 (1.17, 3.22) | 1.10 (0.62, 1.97) | 1.01 (0.45, 2.26) | 1.49 (0.74, 3.01) | 1.54 (0.64, 3.70) |
| Age | 1.08 (1.06, 1.09) | 1.07 (1.06, 1.09) | 1.08 (1.06, 1.11) | 1.07 (1.04, 1.09) | 1.07 (1.05, 1.09) | 1.06 (1.04, 1.08) | 1.10 (1.08, 1.12) | 1.05 (1.02, 1.07) |
| Gender (ref: women) | 1.65 (1.39, 1.97) | 1.66 (1.37, 2.02) | 1.95 (1.39, 2.73) | 1.95 (1.46, 2.61) | 1.32 (1.00, 1.74) | 1.51 (1.18, 1.94) | 1.74 (1.44, 2.10) | 2.13 (1.47, 3.08) |
| Education (ref: none) |  |  |  |  |  |  |  |  |
| Any | 0.86 (0.55, 1.33) | 1.42 (1.10, 1.82) | 0.92 (0.49, 1.73) | 0.97 (0.60, 1.59) | 0.77 (0.56, 1.05) | 1.86 (1.00, 3.45) | 0.62 (0.45, 0.85) | 0.84 (0.54, 1.31) |
| Primary | 1.05 (0.68, 1.63) | 1.10 (0.79, 1.54) | 0.65 (0.36, 1.16) | 0.64 (0.39, 1.04) | 0.75 (0.47, 1.19) | 1.73 (0.93, 3.23) | 0.81 (0.64, 1.02) | 0.77 (0.47, 1.28) |
| Secondary | 0.81 (0.51, 1.28) | 1.34 (0.85, 2.12) | 0.52 (0.27, 1.01) | 0.67 (0.34, 1.30) | 0.61 (0.29, 1.28) | 1.21 (0.64, 2.30) | 0.59 (0.45, 0.79) | 0.63 (0.28, 1.38) |
| Tertiary | 0.94 (0.58, 1.52) | 0.87 (0.48, 1.58) | 0.54 (0.26, 1.14) | 0.75 (0.32, 1.75) | 1.12 (0.57, 2.18) | 1.09 (0.56, 2.13) | 0.50 (0.34, 0.74) | 0.67 (0.22, 2.02) |
| Physical impairment (ref: none) |  |  |  |  |  |  |  |  |
| 1-2 | 1.20 (1.00, 1.44) | 0.79 (0.63, 1.00) | 1.08 (0.75, 1.56) | 1.49 (1.04, 2.13) | 1.09 (0.79, 1.50) | 1.21 (0.92, 1.60) | 1.05 (0.86, 1.28) | 1.35 (0.94, 1.92 |
| 3+ | 1.69 (1.30, 2.18) | 1.12 (0.86, 1.47) | 1.30 (0.83, 2.03) | 1.36 (0.90, 2.06) | 1.56 (1.07, 2.26) | 1.54 (1.11, 2.14) | 1.03 (0.77, 1.38) | 0.87 (0.36, 2.12) |
| Dementia (ref: no) | 2.90 (2.35, 3.58) | 1.84 (1.42, 2.38) | 3.21 (2.13, 4.83) | 1.72 (1.08, 2.75) | 1.68 (1.14, 2.48) | 3.60 (2.64, 4.92) | 2.90 (2.19, 3.84) | 2.09 (1.18, 3.68) |
| Food insecurity (ref: no) | 0.98 (0.66, 1.45) | 0.94 (0.70, 1.26) | 1.62 (0.97, 2.71) | 1.03 (0.55, 1.93) | 1.13 (0.67, 1.91) | 0.81 (0.34, 1.97) | 1.18 (0.36, 3.90) | 0.95 (0.59, 1.52) |
| Assets | 0.90 (0.83, 0.98) | 0.86 (0.81, 0.91) | 1.05 (0.91, 1.22) | 1.05 (0.91, 1.21) | 0.97 (0.89, 1.05) | 0.77 (0.66, 0.91) | 1.00 (0.92, 1.09) | 1.07 (0.93, 1.23) |

**Table S2:** Fully adjusted model of the association between anxiety and mortality

|  | Cuba | Dominican Republic | Peru | Venezuela | Mexico | Puerto Rico | China | India |
| --- | --- | --- | --- | --- | --- | --- | --- | --- |
| Anxiety (ref: none) |  |  |  |  |  |  |  |  |
| Sub-threshold | 1.10 (0.92, 1.31) | 1.25 (1.01, 1.55) | 0.96 (0.68, 1.35) | 1.38 (0.98, 1.92) | 1.47 (1.10, 1.97) | 1.06 (0.83, 1.37) | 1.68 (1.29, 2.18) | 1.77 (1.25, 2.50) |
| Case | 1.05 (0.70, 1.58) | 1.31 (0.92, 1.88) | 0.47 (0.21, 1.06) | 1.91 (1.11, 3.27) | 0.81 (0.39, 1.68) | 1.03 (0.49, 2.16) | 4.07 (1.39, 11.89) | 1.12 (0.30, 4.14) |
| Age | 1.08 (1.06, 1.09) | 1.07 (1.06, 1.09) | 1.08 (1.06, 1.11) | 1.06 (1.04, 1.09) | 1.07 (1.05, 1.09) | 1.07 (1.05, 1.09) | 1.10 (1.09, 1.12) | 1.05 (1.02, 1.07) |
| Gender (ref: women) | 1.63 (1.37, 1.94) | 1.66 (1.37, 2.01) | 1.98 (1.40, 2.78) | 2.03 (1.52, 2.72) | 1.37 (1.04, 1.81) | 1.54 (1.20, 1.96) | 1.80 (1.49, 2.17) | 2.13 (1.48, 3.06) |
| Education (ref: none) |  |  |  |  |  |  |  |  |
| Any | 0.86 (0.55, 1.33) | 1.41 (1.10, 1.81) | 0.88 (0.47, 1.65) | 0.98 (0.60, 1.60) | 0.77 (0.57, 1.05) | 1.89 (1.02, 3.52) | 0.57 (0.42, 0.78) | 0.83 (0.53, 1.29) |
| Primary | 1.05 (0.67, 1.62) | 1.10 (0.78, 1.53) | 0.65 (0.36, 1.15) | 0.63 (0.38, 1.03) | 0.77 (0.48, 1.21) | 1.69 (0.90, 3.16) | 0.78 (0.62, 0.98) | 0.80 (0.48, 1.32) |
| Secondary | 0.79 (0.50, 1.26) | 1.33 (0.84, 2.10) | 0.52 (0.27, 0.99) | 0.64 (0.33, 1.25) | 0.64 (0.31, 1.33) | 1.23 (0.65, 2.33) | 0.56 (0.42, 0.75) | 0.65 (0.30, 1.44) |
| Tertiary | 0.93 (0.57, 1.51) | 0.87 (0.48, 1.58) | 0.53 (0.26, 1.08) | 0.77 (0.33, 1.79) | 1.21 (0.62, 2.35) | 1.12 (0.57, 2.20) | 0.48 (0.33, 0.71) | 0.63 (0.21, 1.85) |
| Physical impairment (ref: none) |  |  |  |  |  |  |  |  |
| 1-2 | 1.22 (1.02, 1.47) | 0.79 (0.62, 0.99) | 1.11 (0.77, 1.59) | 1.44 (1.01, 2.05) | 1.03 (0.75, 1.43) | 1.18 (0.89, 1.56) | 1.07 (0.88, 1.30) | 1.40 (0.99, 1.99) |
| 3+ | 1.78 (1.37, 2.31) | 1.14 (0.87, 1.49) | 1.40 (0.89, 2.21) | 1.29 (0.85, 1.94) | 1.43 (0.98, 2.09) | 1.44 (1.04, 2.00) | 1.00 (0.75, 1.33) | 0.87 (0.35, 2.13) |
| Dementia (ref: no) | 2.91 (2.35, 3.61) | 1.81 (1.40, 2.34) | 3.46 (2.29, 5.22) | 1.63 (1.00, 2.64) | 1.68 (1.14, 2.47) | 3.53 (2.60, 4.81) | 2.72 (2.07, 3.57) | 2.07 (1.20, 3.57) |
| Food insecurity (ref: no) | 0.99 (0.67, 1.47) | 0.94 (0.71, 1.26) | 1.69 (1.02, 2.82) | 1.00 (0.54, 1.84) | 1.08 (0.64, 1.84) | 0.83 (0.36, 1.92) | 1.06 (0.36, 3.11) | 1.00 (0.64, 1.58) |
| Assets | 0.90 (0.83, 0.98) | 0.86 (0.81, 0.91) | 1.05 (0.91, 1.22) | 1.05 (0.92, 1.20) | 0.97 (0.90, 1.06) | 0.76 (0.65, 0.90) | 1.00 (0.93, 1.09) | 1.09 (0.95, 1.26) |

**Table S3:** Fully adjusted model of the association between depression, anxiety and mortality

|  | Cuba | Dominican Republic | Peru | Venezuela | Mexico | Puerto Rico | China | India |
| --- | --- | --- | --- | --- | --- | --- | --- | --- |
| (ref: none) |  |  |  |  |  |  |  |  |
| Depression alone | 1.34 (0.80, 2.24) | 0.65 (0.26, 1.63) | 1.45 (0.42, 5.00) | 0.87 (0.22, 3.38) | 1.48 (0.61, 3.57) | 0.38 (0.04, 3.75) | 0.75 (0.23, 2.49) | 1.24 (0.61, 2.53) |
| Anxiety alone | 1.01 (0.83, 1.23) | 1.12 (0.89, 1.42) | 0.96 (0.65, 1.40) | 1.36 (0.96, 1.92) | 1.63 (1.20, 2.21) | 1.20 (0.92, 1.56) | 1.70 (1.29, 2.25) | 1.68 (1.11, 2.52) |
| Co-morbidity | 1.35 (1.07, 1.71) | 1.42 (1.10, 1.84) | 0.79 (0.49, 1.26) | 1.61 (1.02, 2.54) | 1.06 (0.68, 1.63) | 0.68 (0.42, 1.11) | 1.67 (0.98, 2.84) | 1.99 (1.21, 3.27) |
| Age | 1.08 (1.06, 1.09) | 1.07 (1.06, 1.09) | 1.08 (1.06, 1.11) | 1.07 (1.04, 1.09) | 1.07 (1.05, 1.09) | 1.06 (1.05, 1.08) | 1.10 (1.09, 1.12) | 1.05 (1.02, 1.07) |
| Gender (ref: women) | 1.65 (1.39, 1.98) | 1.67 (1.37, 2.02) | 2.02 (1.43, 2.85) | 2.02 (1.51, 2.72) | 1.37 (1.04, 1.80) | 1.52 (1.19, 1.94) | 1.79 (1.48, 2.17) | 2.17 (1.51, 3.13) |
| Education (ref: none) |  |  |  |  |  |  |  |  |
| Any | 0.86 (0.55, 1.32) | 1.41 (1.10, 1.81) | 0.90 (0.48, 1.68) | 0.98 (0.60, 1.61) | 0.77 (0.56, 1.05) | 1.74 (0.94, 3.23) | 0.58 (0.43, 0.79) | 0.84 (0.54, 1.31) |
| Primary | 1.05 (0.68, 1.62) | 1.10 (0.79, 1.54) | 0.63 (0.35, 1.13) | 0.63 (0.39, 1.04) | 0.77 (0.49, 1.21) | 1.63 (0.87, 3.05) | 0.78 (0.62, 0.98) | 0.79 (0.48, 1.31) |
| Secondary | 0.80 (0.51, 1.27) | 1.35 (0.85, 2.13) | 0.51 (0.26, 0.98) | 0.66 (0.34, 1.28) | 0.64 (0.31, 1.32) | 1.13 (0.60, 2.15) | 0.56 (0.42, 0.75) | 0.66 (0.30, 1.47) |
| Tertiary | 0.94 (0.58, 1.51) | 0.88 (0.49, 1.60) | 0.52 (0.25, 1.07) | 0.80 (0.35, 1.86) | 1.22 (0.63, 2.38) | 1.04 (0.54, 2.03) | 0.48 (0.33, 0.71) | 0.65 (0.22, 1.95) |
| Physical impairment (ref: none) |  |  |  |  |  |  |  |  |
| 1-2 | 1.20 (1.00, 1.44) | 0.78 (0.62, 0.99) | 1.09 (0.75, 1.57) | 1.45 (1.02, 2.07) | 1.04 (0.75, 1.43) | 1.17 (0.88, 1.56) | 1.07 (0.88, 1.31) | 1.38 (0.97, 1.97) |
| 3+ | 1.70 (1.31, 2.21) | 1.10 (0.84, 1.44) | 1.37 (0.87, 2.16) | 1.33 (0.88, 2.00) | 1.45 (0.99, 2.12) | 1.50 (1.08, 2.08) | 1.01 (0.76, 1.35) | 0.83 (0.33, 2.05) |
| Dementia (ref: no) | 2.91 (2.35, 3.61) | 1.81 (1.40, 2.34) | 3.37 (2.25, 5.05) | 1.65 (1.02, 2.68) | 1.66 (1.13, 2.44) | 3.64 (2.68, 4.95) | 2.74 (2.09, 3.60) | 1.94 (1.10, 3.45) |
| Food insecurity (ref: no) | 0.97 (0.65, 1.44) | 0.93 (0.70, 1.24) | 1.69 (1.01, 2.82) | 0.97 (0.52, 1.81) | 1.11 (0.65, 1.87) | 0.85 (0.35, 2.01) | 1.06 (0.36, 3.09) | 0.97 (0.61, 1.54) |
| Assets | 0.91 (0.83, 0.98) | 0.86 (0.81, 0.92) | 1.05 (0.90, 1.22) | 1.04 (0.91, 1.19) | 0.97 (0.89, 1.05) | 0.77 (0.65, 0.91) | 1.01 (0.93, 1.09) | 1.09 (0.95, 1.26) |
